# Unraveling Mammalian Biodiversity in a Non-Protected Area in Tibet: Community Diversity, Species Interactions and Conservation Imperatives

**DOI:** 10.3390/biology15110862

**Published:** 2026-05-30

**Authors:** Keji Guo, Zijun Tang, Ming Su, Tong Zhang, Fu Shu, Qi Li, Haochun Chen, Changjian Wang, Mengfei Zhang, Yang Yu, Yi Chen, Muhammad Zaman, Zuofu Xiang

**Affiliations:** 1 College of Life-Science and Technology, Central South University of Forestry and Technology, Changsha 410004, China; guokeji@126.com (K.G.); wangchangjian1949@163.com (C.W.); yuyangjcl@126.com (Y.Y.); ychen1009@163.com (Y.C.); 2 Central South Inventory and Planning Institute of National Forestry and Grassland Administration, Changsha 410014, China; 13548615578@163.com (Z.T.); 18773155712@163.com (M.S.); zhangtong0429@163.com (T.Z.); shufu328qs@163.com (F.S.); li_qi09@163.com (Q.L.); chenhc_zny@126.com (H.C.); 18774893869@163.com (M.Z.); 3 College of Forestry, Central South University of Forestry and Technology, Changsha 410004, China; wildboyzamu@hotmail.com; 4 Hainan Institute of National Park, 110 Haifu Road, Meilan District, Haikou 570203, China

**Keywords:** camera traps, biodiversity, inter-specific interactions, habitat factors, mammalian communities

## Abstract

Human disturbances, such as habitat destruction and overharvesting, are greatly harming ecosystems and causing significant declines in biodiversity. Although protected areas play a crucial role in conserving terrestrial mammals, nearly non-protected areas (N-PAs) have similar functions, harbor high biodiversity and ecosystem integrity, and deserve to be protected. To identify the conservation value of mammalian species in critical ecosystems within N-PAs, we conducted a camera-trap survey in Luolong County, Tibet, from November 2019 to June 2023, monitoring 159 sites and documenting 25 mammalian species across 28 similar or dissimilar habitats. We found this area was an integrity ecosystem with higher species richness and diversity in scrub and evergreen forests with notable occurrence of herbivores comprising musk deer, chinese serow, woolly hares and carnivores such as the common leopard, snow leopards, red foxes and stone marten. Mammalian species occurrence increased away from human activities. Different habitats and seasons influenced diversity and species interactions. Key findings include species preferences for specific habitats, such as blue sheep on southern slopes during snow, musk deer in mixed forests, and red foxes avoiding alpine meadows. Habitat type, elevation, and human disturbance significantly impacted species distribution and behavior. The study also found that snow leopard activity time negatively correlates with woolly hare, while common leopards are influenced positively by hares and negatively by brown bears. Red foxes are slightly more active near the chinese serow occurrence areas. Other predators and prey, such as eurasian lynx, gray wolves, musk deer and stone martens show specific seasonal and interspecific interactions, with some relationships explaining small portions of variation. Overall, species temporal detection events are interconnected through complex ecological interactions. These findings improve our understanding of habitat hosting for rare species and the balance of endangered prey and predator communities in N-PAs in Tibet, emphasizing their significance for conservation efforts.

## Simple Summary

Human activities such as habitat destruction and overgrazing are significantly destroying ecosystems and triggering significant declines in mammalian biodiversity. While protected areas perform a vital role in maintaining terrestrial mammals, some non-protected areas (N-PAs) have a related role in maintaining high biodiversity and ecosystem integrity. It is essential to determine the conservation value of mammalian species in those critical ecosystems within N-PAs. In these studies, we provide a camera-trap survey in Luolong County, Tibet N-PAs, which has potential for biodiversity conservation. We found that this area was an integrity ecosystem with higher species richness and diversity in scrub and evergreen forests, with the occurrence of important species such as herbivores, including musk deer, chinese serow and woolly hares, and carnivores, common leopards, snow leopards, red foxes and stone martens. Also, these species temporal detection events are interconnected through environmental factors. These results expand our understanding of habitat quality for rare species and the balance of endangered prey and predator communities in N-PAs in Tibet, highlighting their importance for conservation efforts.

## 1. Introduction

In the Anthropocene, human activities are significantly impacting ecosystems, leading to a decline in vital services [1]. Unsustainable practices, such as habitat destruction, invasive species, and over-exploitation, are driving widespread biodiversity loss, especially among rare wildlife populations [2]. Protected areas (PAs) play a crucial role in conserving terrestrial mammals and overall biodiversity [3]. Generally, mammalian species support key ecosystem functions [4] and often serve as umbrella species [5], highlighting the importance of understanding their status—especially as deforestation accelerates worldwide [6]. Degraded or disturbed forests tend to support fewer mammal species, indicating reduced community diversity [7]. Furthermore, recent research has emphasized species richness, persistence, and colonization rates to understand diversity patterns [8], while also examining functional traits like diet and body size as indicators of community health and responses to environmental change [9,10]. These traits help reveal how animals adapt to their roles within ecosystems [11], aiding in the assessment of community vulnerability and resilience [12]. Moreover, studies show that mammal communities in similar habitats tend to exhibit consistent functional trait profiles and respond uniformly to environmental pressures, despite differences in species composition [13].

The Tibetan Plateau, often called the “Roof of the World”, is vital for its unique and delicate ecosystems [14,15]. It hosts a diverse range of mammals, including many endemic and endangered species [15]. Its varied terrain and extreme climate create a variety of habitats that support rich mammalian biodiversity [16]. This region’s mammal community includes notable endangered and near-threatened species such as the snow leopard (*Panthera uncia*) and common leopard (*Panthera pardus*), highlighting its ecological significance [17]. Mammalian species play vital roles in ecosystems, from top predators regulating prey populations to herbivores influencing vegetation [18]. Their interactions—such as snow leopards preying on blue sheep or wolves on marmots—are complex and shape ecosystem structure and behavior [19]. These relationships affect not only population dynamics but also species behavior, distribution, and evolution [20], with top predators impacting prey feeding habits and vegetation patterns [21]. Resource competition further influences community dynamics [22,23], prompting species to adapt to different ecological niches to minimize overlap and spatial-temporal interactions [24]. Understanding these interactions is crucial for predicting responses to environmental changes and guarding conservation efforts [25].

Conservation of mammalian biodiversity on the Tibetan Plateau is increasingly challenged by habitat destruction caused by human activities such as infrastructure development, overgrazing, and mining [26,27]. Expanding settlements and roads not only damage habitats but also increase human access, leading to further disturbances and wildlife exploitation [28]. Climate change further threatens the region by altering temperature, precipitation, and snow cover, which impact species distribution and resource availability [18,29]. Additionally, free-ranging livestock compete with wild animals for resources and can transmit diseases, while human-wildlife conflicts, especially involving carnivores preying on livestock, often result in retaliatory killings that endanger species [27,30].

Therefore, to identify species distribution and abundance patterns in a critical ecosystem known for its rich biodiversity and support future conservation initiatives [31] by using camera-trap data, we conducted research in Luolong County, Tibet. Firstly, we hypothesize that environmental factors such as proximity to water, habitat type, slope, aspect, elevation, vegetation indices, temperature, and snow cover shape species interactions and habitat preferences in non-protected areas (N-PAs) [32,33]. Secondly, we hypothesize that regions with high mammalian diversity and complex interactions may be more vulnerable to human disturbances with nearby settlements and roads accelerating habitat degradation and increasing threatening biodiversity [22,34,35]. Lastly, we identify major conservation challenges influenced by human activities and climate change, and try to develop science-based recommendations to improve conservation strategies, fostering better protection for wildlife and their habitats while encouraging sustainable coexistence with local communities.

## 2. Materials and Methods

### 2.1. Study Area

The study was conducted in the southwestern area of Qamdo, Luolong County (95°10′–95°50′ E, 30°10′–31°50′ N), southeastern Tibet. The region lies along the upper reaches of the Nujiang River and borders the northern segment of the Hengduan Mountains, with its western boundary adjoining the southeastern tip of the Nyainqêntanglha Mountains. The terrain’s average elevation is approximately 3700 m above sea level, covering an area close to 8108 km^2^. The local economy primarily relies on agriculture and livestock farming, cultivating crops such as wheat, highland barley, and rapeseed, while rearing animals like yaks, goats, and pigs [17]. Human activities are mostly assembled in residential zones and along roads, where grazing and harvesting of medicinal fungi like caterpillar fungus (*Ophiocordyceps sinensis*) are common [17].

The climate in this area is classified as a temperate semi-arid monsoon climate, typical of the southeastern Tibetan Plateau. It features abundant sunlight and a significant temperature variation with dry winters and mild summers. The average annual temperature hovers around 5.5 °C, with recorded extremes reaching a maximum of 30.6 °C and a minimum of −22.1 °C. Precipitation averages 424.6 mm annually, predominantly falling between May and September. The region receives over 2500 h of sunshine each year, with a frost-free period of approximately 120 days.

**Figure 1:**
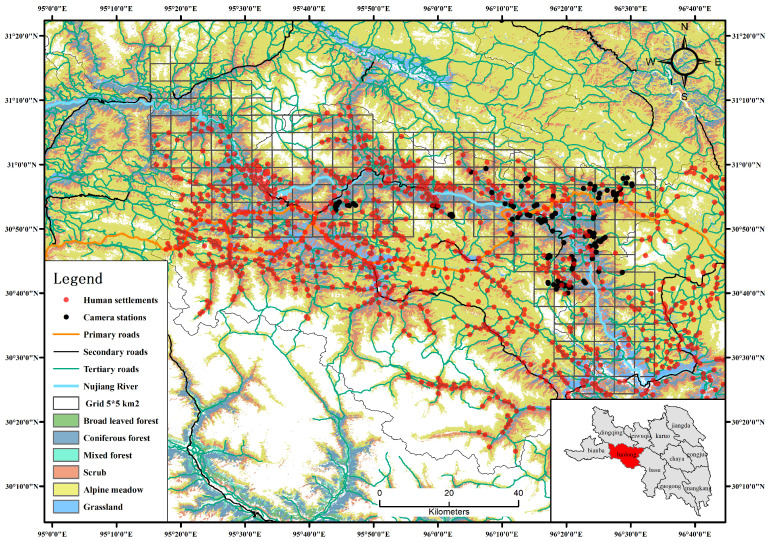
Map of Luolong County, Tibet, showing camera-trap stations, habitat types, and roads in the study zone.

Biodiversity in Luolong County is exceptionally diverse encompassing approximately 25 species of mammals both large and small [36]. Globally important species include white lipped deer (*Cervus albirostris*), common leopard (*Panthera pardus*), leopard cat (*Prionailurus bengalensis*), red dog or dhole (*Cuon alpinus*), red fox (*Vulpes vulpes*), pika (*Ochotona curzoniae*), asian badger (*Meles leucurus*), yellow-throated marten (*Martes flavigula*), himalayan weasel (*Mustela sibirica*), woolly hare (*Lepus oiostolus*), gray wolf (*Canis lupus*), alpine musk deer (*Moschus chrysogaster*), rhesus macaque (*Macaca mulatta*), Eurasian lynx (*Lynx lynx*), stone marten (*Martes foina*), mountain weasel (*Mustela altaica*), Himalayan marmot (*Marmota himalayana*), snow leopard (*Panthera uncia*), blue sheep (*Pseudois nayaur*), wild boar (*Sus scrofa*), Tibetan fox (*Vulpes ferrilata*), hog badger (*Arctonyx collaris*), chinese serow (*Capricornis milneedwardsii*) and yellow-billed weasel (*Mustela sibirica*), and brown bear (*Ursus arctos*). The region’s rich fauna underscores its ecological significance and diversity. Vegetation types encompass coniferous forests, shrublands, and meadows. Coniferous stands are predominantly composed of *Picea likiangensis* and *Abies georgei*. The shrublands primarily consist of species like *Caragana* spp., *Berberis* spp., *Rhododendron* spp., and *Spiraea* spp., while the meadows are primarily dominated by *Carex* species, see Figure 1. Mammalian species and their status in China are presented in Table S1.

### 2.2. Data Collection

#### 2.2.1. Camera-Trap Stations

We used a camera-trapping method to monitor mammalian species temporal detection rates and co-occurrences [37,38]. We created a 5 × 5 km grid system, and camera traps were strategically placed within each cell. In addition, camera-trap placement was adjusted based on field conditions, such as grids with overgrazing had fewer cameras, while some grid areas had 2–4 extra cameras with higher occurrences of mammalian species with less human disturbances, resulting in a semi-systematic survey method and higher detection rates [39,40]. No baits or lures were used to attract animals in the camera stations. Each specific camera operated for roughly six months (180 days), maintaining stable performance for 4 to 5 months, and was permanently fixed at its respective locations and did not change positions. These cameras operated continuously without any delay between consecutive captures. A duplicate record in this study refers to multiple repeated or clustered images/sequences registered of the same individual (or conspecific group of individuals) taken by the same camera-trap station, within a distinct time threshold, with no significant spatial or behavioral separation revealing an independent detection event. Specifically, records were categorized as duplicates if they met all of the subsequent criteria: (1) captured by the same camera-trap unit at a permanent sampling station; (2) recognized as the same species via visual species identification; (3) created within a pre-determined time interval that eliminates frequent detections of the same individual/group due to continuous presence at the camera field-of-view.

We collected data from November 2019 and June 2023 by using 159 camera sites, which were combined into 28 distinct locations covering an area of approximately 1000 km^2^ across elevations from 1878 to 3138 m based on similar/dissimilar landscape features. Two or four cameras were placed at various sites within the same grid with some facing each other to capture images from both perspectives, typically mounted on rocks or tree trunks approximately 50 cm above the ground. Each photo was labelled with metadata including date, time, moon phase, temperature, and habitat type [41]. We used Bolym BG962-X36W and SG2060-K camera traps (Bolymedia Communications, Shenzhen, China), capable of recording color images and videos during the day and monochrome images and videos at night with a trigger speed of 0.20 s.

#### 2.2.2. Gathering Data on Mammalian Diversity Within Community Settings

All raw camera-trap images and videos were imported into Camerabase 2.0, and invalid records (blank images, false triggers from vegetation, light, or camera errors) were manually removed. Valid records were noted with key metadata: camera station ID, specific sampling time, species identity, and individual detection event. Species-specific time thresholds defined independent detections: 30 min for solitary or either mobile species and 15 min for group-living or sedentary species. Using camera-based algorithms, records were grouped by station and species, arranged chronologically, and automatically flagged as duplicates if they fell within the set threshold after the initial detection. Potential duplicates were manually tested by three researchers, with inconsistencies fixed by a senior researcher to account for unusual animal behavior. We confirmed duplicates were detached, retaining only the first record of each independent event. A 10% random quality check confirmed error-free filtering, regulating the workflow to confirm data consistency for ecological examination.

We documented the independent detection events of each mammalian species, noting whether snow was present or absent. Videos featuring humans, livestock, or unidentifiable subjects were excluded from final analysis [22]. We recorded the number of independent detection events for each species captured by the cameras to assess species diversity and accumulation, adhering to the methodology outlined by [42,43]. The sampling effort was calculated by multiplying the total number of camera-traps by the sampling duration in days. Each species detection rate within a 24-h period was treated as a separate event, with multiple individuals in a group counted individually such as wolves, blue sheep etc. [44]. We categorized habitat types into various classes, including alpine meadows or grasslands, evergreen coniferous forests, mixed forests (comprising broadleaf and needle leaf species), and scrub or shrub land areas using global vegetation cover data obtained from the website (https://data.tpdc.ac.cn) [39].

Moreover, we extracted information on the proximity distance to water bodies (m) (such as rivers and streams), as well as the nearest roads (m) and human settlements or grazing areas(m) from the source (www.webmap.cn) [45]. Topographical features like slope, aspect, and elevation (m) were derived using digital elevation models (DEM) available from free online resources at (https://www.gscloud.cn) [46]. Normalized Difference Vegetation Index (NDVI) of the study area was also downloaded from (https://data.tpdc.ac.cn) [18]. Temperature data were obtained from camera-trap sources, while mean annual precipitation was downloaded from (https://www.worldclim.org). All these environmental variables were measured and integrated into the analysis using ArcGIS 10.2.

#### 2.2.3. Interactions Within Mammalian Communities and Their Association with Habitat Resources

We assessed temporal detection rates for each species, including both predator-prey pairs, and organized data into separate Excel sheets for carnivores and herbivores [47]. The data were further categorized based on camera sites, with conditions combining snow presence or absence. Furthermore, habitat factors associated with each species were documented; species with very low detection rates (fewer than 30 detections) were excluded from final habitat preference analyses (such as wild boar, etc.). We also analyzed the 24-h activity patterns of each species using timestamps from camera traps, which were aligned with Tibetan local time [48]. We calculated the activity patterns time of mammalian species based on the timing of recorded videos, which were timestamped with both date and camera ID: (1) nocturnal, characterized by activities primarily occurring from one hour after sunset until one hour before sunrise; (2) diurnal, occurring mainly between one hour after sunrise and one hour before sunset; and (3) crepuscular, encompassing periods from one hour before sunrise to one hour after sunrise, and from one hour before sunset to one hour after sunset [19] and combined into one data set like activity times.

### 2.3. Data Analysis

#### 2.3.1. Species Community Diversity and Accumulation

To evaluate whether the number of survey sites was sufficient for detecting species richness, we pooled data from all cameras. We then analyzed species richness and accumulation patterns based on observations at camera placement within different study sites. Observed richness was estimated using the incidence of rare species following [49]. We used community diversity function methods and quantified with Hill numbers, where q = 0 reflects species richness, q = 1 corresponds to Shannon diversity, and q = 2 indicates Simpson diversity in R package iNEXT (v3.0.2). Rarefaction curves comparing these Hill-based diversity indices were also generated for each camera placement feature. As q increases, the influence of rare species on diversity estimates decreases [49]. Community diversity was assessed through four components: species richness, abundance, diversity indices, and trophic guilds [44], across four habitat types differentiated by the presence or absence of snow. Species richness was measured as the total number of species detected via camera traps. Camera-trap diversity was calculated using the formula [50]:

D = (∑S_i = 1, pi2)

where S is the number of species at each site, and pi is the proportion of each species detection at that site.

Shannon and Simpson indices were employed to evaluate diversity, accounting for both species richness and evenness, complemented by accumulation curves [49]. Species rarefaction curves were constructed to compare species richness and detection rates across sites [44,49]. Confidence intervals (95%) were estimated using the unconditional variance method with 1000 permutations, following [42]. Moreover, non-parametric Mann–Whitney U-tests were conducted to compare species richness among habitat types and snow conditions, with *p*-values adjusted for multiple testing and significance determined accordingly [37]. Data analyses were performed using R packages (v 4.4.1) including *iNEXT*(v3.0.2) for diversity and rarefaction analyses, along with *dplyr* (v1.1.4), *tidyr* (v1.3.1), *ggplot2* (v3.5.1), *tibble* (v3.2.1), and *patchwork* (v1.3.0) for data manipulation and visualization.

#### 2.3.2. Community Guild Associated with Habitat Resources

To evaluate potential overdispersion across all global models, we initially employed QQ plots and residual plots using the GGally package (v2.2.0) [37]. We then developed 16 linear mixed-effects models (LMMs) with the *lme4* (v1.1-35.5) and *ggplot2* packages (v3.5.1) to explore the relationship between each mammalian species and habitat variables, incorporating camera ID as a random effect. Prior to modeling, correlation analysis was performed to exclude variables with coefficients above 0.7, minimizing multicollinearity; effect sizes were calculated using the “mvnormalTest” package (v1.0.1). Temporal detection rates of each independent species served as response variables, with habitat factors as fixed effects. Model selection was determined based on the corrected AIC (*AICc*), delta AICc (*ΔAICc*), and model weights using the AICcmodavg package (v2.3-3) in R [51]. Significance of parameters was evaluated through their estimated coefficients, standard errors (*SE*), and 95% confidence intervals (*CIs*).

#### 2.3.3. Interspecific Interactions Within Carnivores, Herbivores and Omnivores

We examined the interactions among species using linear regression models (LMs). The activity times for each species over a 24 h period were selected as response variables for a particular target species, with the activity times of other species serving as predictor factors. We also accounted for multicollinearity, and it was found that the activity time of macaques caused issues in livestock and humans, leading to its removal from the models [52]. The best model was selected based on degrees of freedom, the adjusted R-squared value, and the F-statistic with significance levels set at *p* < 0.05. All analyses were conducted using RStudio version 4.0.5.

## 3. Results

### 3.1. Diversity of Mammalian Communities Across Various Habitats

**Figure 2:**
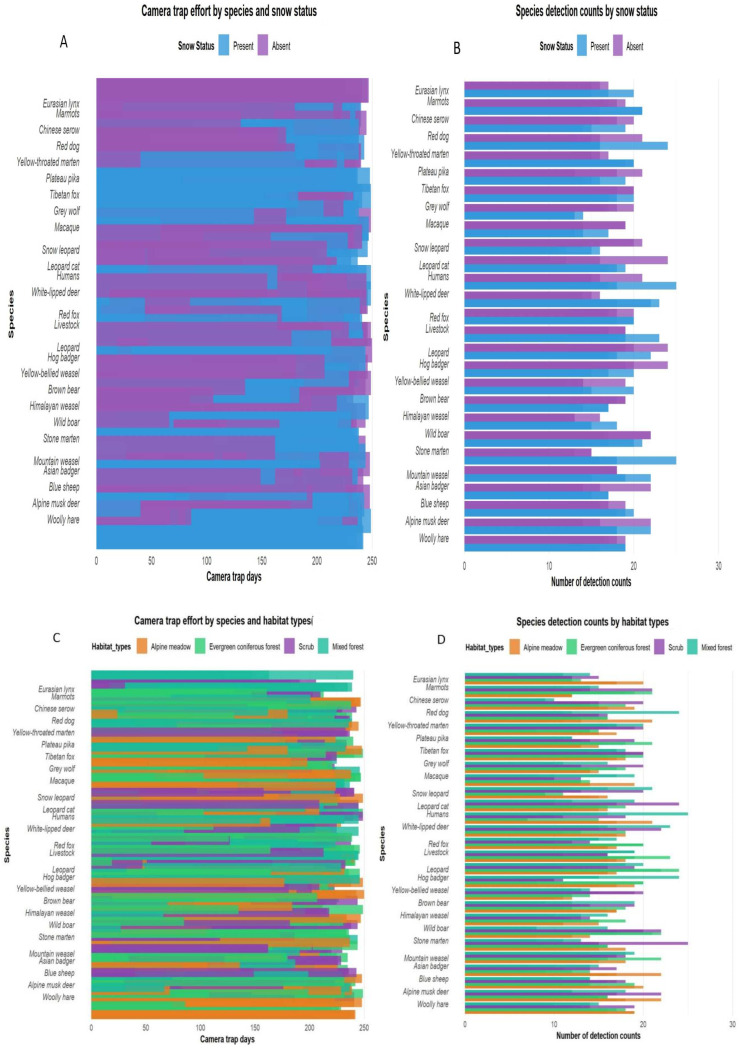
(**A**–**D**): Temporal detection rate of 25 mammalian species across four habitat types (presence vs. absence of snow) using camera-trap data in the Tibetan region.

**Figure 3:**
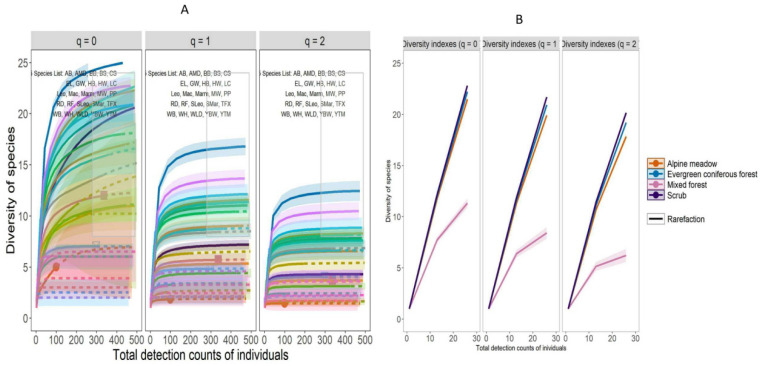
(**A**,**B**), species diversity indices (q = 0: Richness, q = 1: Shannon, q = 2: Simpson) and rarefaction curves for 25 mammalian species across habitat types in Tibetan region (presence vs. Absence of snow) and 9 mammalian species rare detection and (F), species diversity indices (q = 0: Species richness, q = 1: Shannon diversity, q = 2: Simpson diversity) across four habitat types, highlighting higher diversity in evergreen forests and scrub zones compared to mixed forests and alpine meadows (25 mammalian species, Tibetan region). Species list are indicated the detection rate of each species such as (AMD (alpine musk deer), BB (brown bear), LC (leopard cat), Leo (common leopard), GW (grey wolf), Marm (himalayan marmots), Sleo (snow leopard), AB (asian badger), WB (wild boar), WH (woolly hare), WLD (white lipped deer), CS (chinese serow), Smar (stone marten), TFX (Tibetan fox), YBW (Yellow bellied weasel), YTM (yellow throated marten), PP (plateau pika), BS (blue sheep), Mac (macaque), RD (red dog), EL (eurasian lynx), HB (hog badger).

**Figure 4:**
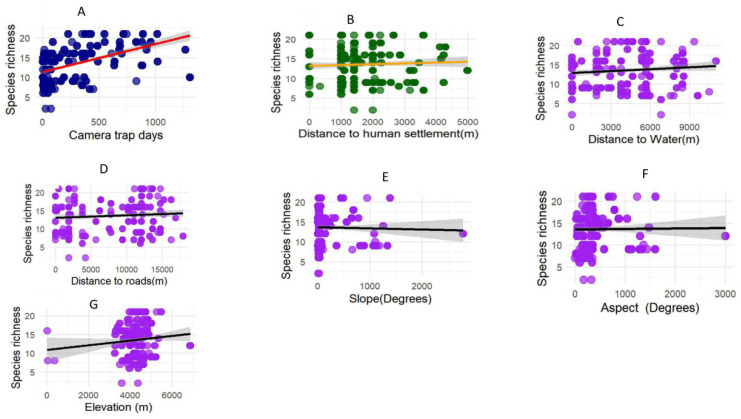
(**A**–**G**): Relationships between species richness and key habitat factors (distance to humans, distance to water, elevation, aspect) (25 mammalian species, Tibetan region).

We collected a total of 10,202 photographic events from the camera traps for all mammalian species and revealed higher species richness and diversity across 28 similar/dissimilar habitats with notable counts for alpine musk deer (990 individuals), chinese serow (742), and woolly hare (830) (Figure 2A–D). Conversely, himalayan marmots, plateau pikas, and white-lipped deer exhibited lower detection events. Among carnivores, the common leopard (1029), red fox (1237), and stone marten (532) showed higher detection rates, while snow leopards, red dogs, mountain weasels, and yellow-billed weasels showed lower counts (Supplementary Tables S2 and S3; Figure 2A,B). We found that scrubs and evergreen coniferous forests demonstrated significantly higher mammal diversity than mixed forests and alpine meadows (*U* = 333.0, *p* < 0.01), whereas differences between the latter habitats were not significant (*U* = 204.0, *p* = 0.120) (Figure 3A,B). We examined that mammalian species abundance and diversity significantly varied between snowy and snow-free days (*U* = 211, *p* < 0.05) (Figure S1). We recorded that species richness increased with distance from human settlements (*R*^2^ = 0.711, *p* < 0.001; Figure 4B), distance from water sources (*R*^2^ = 0.441, *p* = 0.041; Figure 4C), and distance from roads (*R*^2^ = 0.881, *p* < 0.023; Figure 4D), and was highest at moderate elevations (*R*^2^ = 0.721, *p* < 0.021; Figure 4G). The rarefaction curve indicated a low overall diversity with around 25 mammal species identified (Figure 3A). Diversity indices, including Simpson and Shannon, were higher in scrub, coniferous forests, and alpine meadows, but lower in mixed forests (Figure 3B). Furthermore, significant differences in species richness and abundance across habitats are shown in Figure S1C–F.

### 3.2. Resource Selection by Mammalian Community: Both Predators and Preys

#### 3.2.1. Prey Species

The optimal LMMs for blue sheep temporal detection rate revealed a strong link to aspect, particularly the southern aspect during snow conditions, with a camera ID random effect of 0.024 (Table 1 and Table 2). For chinese serow, the best model included NDVI and scrub habitats (*K* = 4), indicating a preference for scrub zones, although NDVI negatively influenced presence (Table 1 and Table 2). Musk deer temporal detection rate was best explained by a model with snow status and habitat types (*K* = 8). Snow days reduced detection, suggesting avoidance of snow covers, and showed a preference for mixed forests. Furthermore, white-lipped deer temporal detection rate was best modelled with two variables (*K* = 7; *AIC_C_* = 163.39), showing avoidance distance from roads and a liking for scrub habitats. Woolly hare temporal detection rates were influenced by three variables (*K* = 8; *AIC_C_* = 4340.50); alpine meadows increased detections, whereas precipitation, evergreen forests, and distance from water sources decreased sightings. Plateau pikas temporal detection rate was best predicted by two variables (*K* = 7; *AIC_C_* = 2573.09), favoring alpine meadows and scrub zones. Rhesus macaque temporal detection rates were primarily affected by moderate elevation and higher NDVI (*K* = 4; *AIC_C_* = 249.70). Lastly, himalayan marmots were best modelled with two variables (*K* = 4; *AIC_C_* = 412.76), with lower elevations negatively impacting detection, while other factors were not significant.

#### 3.2.2. Predators

The temporal detection rate of brown bears was primarily influenced by four factors: temperature, snow cover, slope, and habitat type (*K* = 10; *AIC_C_* = 487.11). Higher temperatures and snow presence negatively impacted detection rates, while bears preferred mixed forests. Grey wolf showed avoidance of roads and human settlements (*K* = 5; *AIC_C_* = 283.62). Badger temporal detection rate was best explained by three variables, notably decreased occurrence with higher temperatures and snow cover (*K* = 6; *AIC_C_* = 136.52). Leopard cats temporal detection rate was explained by one variable, slope, with an inclination to avoid steep areas (*K* = 4; AIC_C_ = 191.33). Eurasian lynx temporal detection rates were driven by two factors: avoiding northern aspects and favoring shrub zones (*K* = 7; *AIC_C_* = 110.57). The common leopard’s temporal detection rate was influenced by three variables; they were more detectable with snow presence but avoided distance from roads (*K* = 6; *AIC_C_* = 385.22). Snow leopard temporal detection rate relied on four factors, favoring mixed and coniferous forests with no clear relation to temperature or altitude (*K* = 10; *AIC_C_* = 1693.88). Red foxes temporal detection rate showed a preference for scrub and avoiding alpine meadows (*K* = 6; *AIC_C_* = 1193.30). Red dogs temporal detection rate was best explained by two variables, mainly avoiding distance to roads (*K* = 7; *AIC_C_* = 82.53). Yellow-billed weasels temporal detection rate was influenced by four factors, tending to avoid rain and favor coniferous forests (*K* = 9; *AIC_C_* = 47.08). Stone martens temporal detection rate was mainly affected by water body presence, which they avoided (*K* = 3; *AIC_C_* = 404.70).

### 3.3. Interaction Among the Community Levels: Both Predators and Preys

#### 3.3.1. Carnivores

The timing of snow leopard activity was primarily negatively linked to that of the woolly hare. The overall model was significant (F_(1, 261)_ = 5.715, *p* < 0.017) with a modest explanation (*R*^2^ = 0.017). The activity of common leopards showed significant interactions with brown bears and woolly hares. The common leopards baseline activity was 0.624 (*SE* = 0.035, *p* < 0.001). Woolly hares showed a positive association (intercept = 0.109, *SE* = 0.044, *p* < 0.013; Figure S2), while brown bears had a negative relationship (intercept = −0.143, *SE* = 0.050, *p* < 0.004; Figure S2). The combined model was significant (F_(2, 260)_ = 6.138, *p <* 0.002) with an adjusted *R^2^* of 0.037.

Red fox activity was positively related to the Chinese serow with intercepts of 0.407 (*SE* = 0.046, *p* < 0.001; Figure S2) and 0.194 (*SE* = 0.086, *p <* 0.025), respectively. The overall model was significant (F_(1, 261)_ = 5.017, *p <* 0.025) and explained a small portion of variance (*R*^2^ = 0.015).

Leopard cat activity timing was negatively associated with yellow-billed weasels (Figure S2h). The intercept was 0.659 (*SE* = 0.037, *p* < 0.001), with the yellow-billed weasels effect near significance (intercept = −0.112, *SE* = 0.058, *p <* 0.056). The model was marginally significant (F_(1, 261)_ = 3.684, *p* < 0.056) with a slight negative correlation (adjusted *R*^2^ = 0.010).

The temporal activity of eurasian lynx negatively correlated with that of woolly hares (intercept = 0.405, *SE* = 0.051, *p* < 0.001; Figure S2p) and positively with white lipped deer (intercept = 0.278, *SE* = 0.077, *p <* 0.003; Figure S2o), with the model explaining about 5.8% of variation (F(_1, 260_) = 9.191, *p* < 0.013; *R*^2^ = 0.058). Similarly, gray wolf activity showed a positive association with white-lipped deer (intercept = 0.363, *SE* = 0.043, *p* < 0.001; Figure S2z), accounting for roughly 1.6% of variation (F_(1, 261)_ = 5.456, *p <* 0.020). Redfox activity was positively linked to musk deer (intercept = 0.488, *SE* = 0.017, *p* < 0.001; Figure S2r) explaining about 1.9% of variance (F_(1, 261)_ = 6.129, *p <* 0.013). Stone marten activity was positively associated with hare (intercept = 3.286, *SE* = 2.703, *p* < 0.001; Figure S2f), contributing around 10.1% to the model (F_(1, 261)_ = 5.092, *p <* 0.003). Lastly, yellow-billed weasel activity was negatively related to leopard cats (intercept = 0.589, *SE* = 0.045, *p* < 0.001), with the model explaining 1.1% of the variance (F_(1, 261)_ = 3.684, *p* < 0.056; Figure S2u).

#### 3.3.2. Temporal Interactions of Herbivores and Omnivores

The temporal activity of himalayan marmots negatively interacted with brown bears (Figure S2d), but showed significant positive associations with stone martens and tibetan pikas (intercept = 3.130, *SE* = 4.749, *p* < 0.001; tibetan pika intercept = 1.399, *SE* = 6.189, *p* < 0.024, Figure S2s); brown bear intercept = −1.089, SE = 4.736, *p* < 0.022; stone marten intercept = 1.000, *SE* = 4.129, *p* < 0.001).

Tibetan pikas exhibited significant positive activity correlation with himalayan marmots (intercept = 0.478, *SE* = 0.024, *p* < 0.001; marmot intercept = 0.089, SE = 0.040, *p* < 0.028; Figure S2a). Woolly hare activity was negatively associated with snow leopards and eurasian lynxes activity was positively related to snow leopards and brown bears (intercept = 0.455, *SE* = 0.089, *p* < 0.001; snow leopard intercept = 0.201, *SE* = 0.084, *p* < 0.017, Figure S2l; snow leopard intercept = −0.142, *SE* = 0.070, *p* < 0.044, Figure S2x; eurasian lynx intercept = −0.151, *SE* = 0.072, *p* = 0.036, Figure S2n; brown bear intercept = 0.201, SE = 0.068, *p* < 0.003, Figure S2k). Brown bear activity was negatively linked to snow leopards and himalayan marmots but positively correlated with woolly hare activity (intercept = 0.622, *SE* = 0.061, *p* < 0.001; snow leopard intercept = −0.214, *SE* = 0.074, *p* < 0.004, Figure S2b; woolly hare intercept = 0.156, *SE* = 0.053, *p* < 0.003; Figure S2e; himalayan marmot intercept = −0.098, SE =0.052; *p* < 0.059; Figure S2c).

The temporal activity of musk deer showed a significant positive association with red dog (intercept = 0.262, *SE* = 0.053, *p* < 0.001) and red dog intercept (0.243, *SE* = 0.098, *p* < 0.013; Figure S2q). Similarly, the activity of the chinese serow was positively correlated with red fox (intercept = 0.418, *SE* = 0.027, *p* < 0.001, Figure S2w; red fox intercept = 0.096, *SE* = 0.043, *p* < *0.025*), with the model significant (F_(1,261)_ = 5.017, *p* < 0.025). White lipped deer activity negatively interacted with himalayan marmots but showed significant positive relationships with eurasian lynx and gray wolves (intercepts = 0.462, *SE* = 0.036, *p<* 0.001;eurasian lynx intercept = 0.142, *SE* = 0.046, *p <* 0.002, Figure S2v; gray wolf intercept = 0.106, *SE* = 0.048, *p* < 0.029; Figure S2y), while the himalayan marmot interaction was negative (−0.108, *SE* = 0.037, *p* <0.007; Figure S2t).

## 4. Discussion

We documented 25 mammalian species across 28 defined similar or dissimilar habitats in Luolong County, southeastern Tibet, and found that this ecosystem was well-integrated with the presence of top predators, mesopredators, and prey species within the different habitats. However, mammalian species exhibited dodging to the nearest human settlements and roads. These findings confirm that human activities and infrastructure construction have diverse effects on diversity and distribution and suggest the declaration of protected areas to serve as vital shelters to secure biodiversity [53]. However, data on biodiversity in high-altitude regions remain limited. In this term, our data imply that the area should be considered as a protected area in the future.

### 4.1. Species Heterogeneity in Different Habitats

Biodiversity assessment plays a crucial role in documenting the status and existence of rare and endangered species [31]. Low levels of biodiversity often indicate reduced redundancy in the functional roles fulfilled by various species, meaning that the loss of key species can have a disproportionately negative impact on ecosystems and the essential services they provide. Establishing baseline biodiversity conditions and continuously monitoring wildlife populations are essential steps in evaluating the success of protected areas and reintroduction efforts [54]. Although red dogs and tibetan foxes display extremely low detection during the camera-trapping periods, our findings suggest that most mammalian species prefer habitats such as coniferous forests, broad-deciduous leaved forests, and scrub zones.

Furthermore, species diversity shows a negative relationship with certain anthropogenic and geographical factors: areas closer to rivers, roads, and human settlements tend to harbor lower diversity. Additionally, most species are distributed within mid- to high-elevation ranges in the study region. For instance, assessments of biodiversity on seasonal or spatial-temporal scales, considering apex predators, mesopredators, and medium to large prey species, indicate habitat quality variations across these domains [47].

We also explored that most mammals tend to occupy areas characterized by low human activity and greater distances from roads, mirroring patterns observed in other studies of mammalian spatial use [55]. This camera-trap survey successfully identified nine mammalian species classified as rare, demonstrating the utility of this method in capturing data on elusive taxa and enhancing biodiversity assessments for the region. Conducting further surveys over larger spatial extents or extended timeframes could help verify the activity patterns observed, deepen our understanding of rare species ecological needs, and support more informed, evidence-based conservation planning for Tibet’s mammalian communities, as highlighted in recent studies in China [56]. These studies also reveal that climate factors, such as snow cover and low temperatures, and grazing pressures, significantly influence species distribution. For example, woolly hares tend to avoid snowy areas, while snow prefers low-NDVI scarp zones.

### 4.2. Species Detection Rate Linked to Habitat Factors

#### 4.2.1. Herbivores and Omnivores

It was found that blue sheep are more likely to be found on south-facing slopes during snow presence days, but this pattern does not hold in snow-absent days. This seasonal shift suggests that slope aspect is a crucial factor in their habitat selection as seen in Tibetan antelopes (*Pantholops hodgsonii*) [57]. The chinese serow shows a clear preference for scrub habitats, which likely offer easy access to food sources. During winter, chinese serow may browse on plant twigs within these scrub zones to meet its dietary needs as detected in the mainland serow (*Capricornis sumatraensis*) within Tangjiahe Natural Reserve in China [58]. Furthermore, musk deer favour mixed forest habitats. They tend to avoid active movement during snowy conditions, as evidenced by reduced detection rates when snow is present [59].

White-lipped deer reveal distinct movement patterns in relation to human infrastructure. They tend to avoid busy roads such as major highways, but occasionally utilize smaller trails like footpaths and livestock routes within forested regions for movement, and showed a difference with former results in China [60].

It is indicated that the woolly hare predominantly inhabits alpine meadows, where it mainly forages and carries out most of its activities. While we occasionally observed groups of two or three hares, most detections involved solitary individuals. Notably, the woolly hare tends to avoid areas with higher rainfall, evergreen mixed forests, and regions close to water sources. Such consistency highlights shared adaptive strategies among hare (*Lepus capensis*) species for efficient foraging and predator avoidance in high-altitude environments [52]. Plateau pika (*Ochotona curzoniae*) is most frequently detected in alpine meadows and scrub zones, highlighting these habitats as key areas for their presence. Pikas were occasionally observed in small groups of 1–2, but were mostly seen alone. Notably, the species is active during daytime hours in the Qinghai-Tibetan Plateau [61].

This indicates that rhesus macaques are more frequently detected in areas with moderate elevation and higher NDVI, suggesting that regions with moderate altitude and abundant vegetation are vital for their survival. These environments likely offer essential food sources, such as fruits and plants, along with suitable shelter to withstand local environmental challenges [62].

We noticed that marmots tend to be detected at the highest elevations, favoring moderate terrain, open meadows, and grasslands. In addition, some individuals along the periphery of shrub zones are predominantly active during the daytime (diurnal), as seen in Nepal [63]. These habitat preferences or activity patterns confirm that moderate, grassy areas are vital for their foraging and survival [64]. Brown bears were less frequently detected during cold weather and snowy conditions, suggesting they tend to avoid harsh winter environments. They prefer mixed forest habitats, with their selection primarily driven by terrain and elevation rather than proximity to human activities such as roads or settlements (Frąckowiak et al., 2014) [65].

#### 4.2.2. Large and Meso-Carnivores

It is indicated that gray wolves generally avoid high-traffic roads and regions near livestock grazing areas, likely as a strategy to minimize human-wolf conflicts, since proximity to settlements, roads, and grazing sites increases the risk of interactions with people. Interestingly, we did observe some exceptions: occasional detections of wolf packs near livestock trails and tracks. Most sightings involved groups of 2 to 3 wolves, with occasional packs numbering up to five individuals. Gray wolves often prefer areas with abundant livestock and may prey on or conflict with humans near settlements [66] and livestock corrals [51].

The leopard cat tends to avoid steep slopes, preferring smaller trails for movement. Conversely, research indicates that their resting sites are typically located in natural habitats characterized by limited visibility, such as areas with shrubs, reeds, and stones, which are also distant from zones with high human activity [67]. The red fox tends to prefer scrub areas while avoiding alpine meadows, roads, and evergreen forests, as roads have a notably negative effect on their habitat selection [68] and preference to herbs or shrubs land in China [58]. The yellow-billed weasel appears to be negatively influenced by precipitation and prefers evergreen forests, likely avoiding rainy seasons to reduce activity during unfavorable conditions. It tends to stay away from water bodies, potentially due to flood risks or other threats. Similar studies suggest that mesopredators often minimize conflicts with top predators and humans across spatial and temporal scales [69]. Additionally, eurasian lynx favor scrub habitats, which offer essential shelter for hiding from predators and hunting small to medium prey such as hares and rodents. This habitat selection underscores the importance of scrub zones for supporting healthy lynx populations in the study area [70]. Snow leopards during the snow cover do not significantly affect their detection rates. This resilience likely stems from their specialized adaptations—such as dense fur and large paws suited for snowy terrain—and their adaptable foraging strategies [34]. The snow leopards tend to avoid roads, highlighting the negative impact of linear infrastructure on their habitat [71].

Common leopards predominantly favor mixed and evergreen coniferous forests, aligning with their adaptable yet forest-dependent nature. These habitats feature intricate vertical structures—dense canopies for concealment, layered understories for hunting prey such as deer and wild boar, and tree hollows ideal for resting or raising cubs [71]. Additionally, compared to open terrains, these forest types provide more stable microclimates, enabling common leopards to remain active and reproduce throughout the year [72]. Red dogs (dhole) tend to avoid roads and evergreen coniferous forests. Their avoidance of roads is likely due to sensitivity to human disturbance, as roads increase exposure to vehicles, livestock, and settlements, which can disrupt their cooperative hunting behaviors that depend on undisturbed environments [73], Similarly, their reluctance to inhabit evergreen coniferous forests may stem from lower prey availability—these forests typically harbor fewer herbivores—and dense vegetation that can hinder their pursuit of prey [9].

### 4.3. Interspecific Interaction Among the Species

The activity time of snow leopards is only linked to the detection of hares, and this relationship is negative. This suggests that hares are not a primary component of their diet, which likely depends more heavily on other prey species [74]. A greater abundance of alternative prey, such as blue sheep, in the study area may have an effect on their activity patterns [24].

The activity patterns of the common leopard are positively linked to hare occurrence, indicating that hares are likely a significant prey item. Additionally, common leopard activity is negatively correlated with brown bears [18]. This probably reflects a strategy to minimize direct conflicts [75] and decreases interspecific conflicts in shared habitats [76].

Red foxes and chinese serow tend to be active simultaneously, indicating they likely coexist without significant competition or avoidance in shared habitats. According to optimal foraging theory, both predators and prey adopt strategies that maximize their survival and reproductive success by balancing energy expenditure with foraging opportunities [77]. The activity patterns of leopard cats tend to be inversely related to those of yellow-billed weasels, likely as a strategy to minimize interspecific conflict. This suggests that these predators either avoid direct interactions or adjust their behaviors to reduce competition [78] and alter their diet choices [79].

Eurasian lynx activity shows a positive correlation with both hare and white-lipped deer’s presence, suggesting their active periods may coincide with times of prey, and eurasian lynx sometimes hunt both small and large prey species [80]. Red fox activity appears to be linked to musk deer movements, while stone marten activity aligns with marmot presence [81]. Additionally, yellow-billed weasel activity was negatively associated with leopard cat activity, indicating potential avoidance behaviors to reduce conflict [48]. Among small carnivores, the stone marten and leopard cat, which are similar in size and diet, may compete when they coexist, especially since both prefer chasing small prey [82].

Himalayan marmot activity tends to decrease in the presence of brown bears, likely as an anti-predator strategy since bears may prey on them [83]. Conversely, marmots are more active near stone martens and plateau pikas, suggesting limited competition and the possibility of coexistence with these species [84]. Hares, on the other hand, reduce their activity when brown bears, common leopards, and eurasian lynx are nearby, likely to avoid predation by these large predators [85]. Woolly hare activity increases around common leopards and bears, which may reflect shared habitat preferences rather than direct threat, or perhaps these predators focus on other prey, making hares relatively safer [18]. Musk deer display increased activity simultaneously with red foxes, indicating they neither avoid nor typically evade these predators—suggesting minimal threat or conflict [86]. Similarly, chinese serow also tend to be active when red foxes are present, implying a lack of avoidance and a possible tolerance towards this predator, unlike their responses to other carnivores [79]. In contrast, white-lipped deer are less active when himalayan marmots are nearby but show heightened activity in the presence of eurasian lynx and gray wolves. This pattern demonstrates that prey animals actively modify their behavior—either seeking or avoiding certain predators—to mitigate risks and improve survival chances [19,76].

## 5. Conservation Implications and Future Research Directions Using Camera Traps

The camera-trap data in this study reveal crucial insights into the habitat preferences, diversity, and interspecies interactions of Tibet’s mammals, providing valuable guidance for conservation efforts and identifying these non-protected areas for future research [87]. Key habitats have been identified for flagship and rare species: mixed forests are vital for musk deer and brown bears; alpine meadows support tibetan pikas and himalayan marmots; scrub zones are essential for chines serow and white-lipped deer, and mid-elevation zones with high NDVI are important for macaques. Moving forward, surveys should focus on mapping these habitats as interconnected corridors rather than isolated patches—for example, tracking snow leopards (which favor small animal trails but avoid busy roads) as they move between high-elevated areas to low-elevated areas in winter [88]. Additionally, understanding whether chinese serows can access winter forage in the scrub zone when nearby alpine meadows are heavily grazed, and this grazing pressure can be reduced through rotational grazing policies to protect critical habitats.

Behavioral observations, such as gray wolves avoiding high-traffic roads and brown bears remaining inactive during the presence of snow or cold temperatures, as well as species that show positive associations like common leopards and woolly hares, can be used to design timing-based conservation strategies. Future research should include seasonal camera-trap deployments to identify periods of heightened human-wildlife conflict—enabling herders to adjust grazing times or deploy deterrents during peak activity periods. Monitoring activity overlaps between predators and prey over multiple years can also reveal how climate change—such as earlier snowmelt—may disrupt these interactions, potentially threatening prey populations and ecosystem stability. Moreover, monitoring smaller carnivores such as stone martens and leopard cats alongside their prey and competitors will shed light on how human activities, like expanding road networks, might disrupt these delicate balances—information vital for safeguarding the smaller species that underpin the entire food web [89]. Furthermore, this approach can test the effectiveness of conservation initiatives; for instance, whether protected areas in scrub zones encourage the return of species like the yellow-billed weasel (which favor evergreen forests) or if reduced logging fosters increased brown bear sightings in mixed forests. Camera traps serve as vital tools—not just for detecting species presence [44], but for unraveling the intricate relationships among the mammalian species and their conservation challenges.

## 6. Conclusions

Our study found that the non-protected area of Luolong County (Tibet) was an intact ecosystem with higher species richness and diversity in scrub and evergreen forests, with an important presence of herbivores like musk deer, chinese serow, and woolly hares. Carnivores like common leopards, snow leopards, foxes, and martens were also commonly detected. Mammalian species occurrence increased away from human activities. We documented that different habitats and seasons influenced diversity and species interactions. Furthermore, each species has preferences for specific habitats, such as blue sheep on southern slopes during snow, musk deer in mixed forests, and red foxes dodging alpine meadows. Large carnivores such as snow leopards’ activity time negatively links with woolly hares, while common leopards showed a positive association with hares and a negative association with brown bears. For mesopredators, such as red foxes, detection was more often at the occurrence of chinese serows. We found that camera traps serve as a vital tool for detecting species presence during the presence or absence of snow periods in mountainous landscapes and unraveling the intricate relationships among mammalian species and their conservation challenges in remote areas of Tibet, China.

## Figures and Tables

**Table 1:** An overview of LMMs with a delta AICc (*∆AICc*) less than 2 for mammalian species habitat resource selections. AICc refers to Akaike’s Information Criterion adjusted for small sample sizes. The column labelled K indicates the number of degrees of freedom, Wt stands for the Akaike weight, and random effects (RE) explained by models.

Models	K	AICc	D_AICc	Wt	Variance	Std. Dev
Bule sheep detection rate ~ aspect + (1|Camera.ID)	4	3304.59	0.000	1	0.024	0.155
Chines serow detection rate ~ NDVI + habitat types + (1|camera.ID)	8	914.70	0.000	1	0.052	0.229
Musk deer detection rate ~ snow status + habitat types + (1|camera.ID)	8	6646.89	0.000	1	0.032	0.179
White lipped deer detection rate ~ roads + habitat types+ (1|camera.ID)	7	163.39	0.00	1	0.021	0.146
Wolly hare detection rate ~ precipitation + habitat types + water + (1|camera.ID)	8	4240.50	0.00	1	0.018	0.136
Tibetan pika detection rate ~ humans + habitat types + (1|camera.ID)	7	2573.09	0.00	1	0.029	0.1730
Rhesus macaque detection rate ~ altitude + NDVI + (1|camera.ID)	4	249.70	0	1	0.03543	0.1882
Himalayan marmots detection rate ~ temperature + altitude + (1|camera.ID)	4	412.76	0	1	0.011	0.108
Brown bear detection rate ~ temperature + snow status + slope + habitat types +(1|camera.ID)	10	487.11	0	0.8	0.054	0.234
Grey wolf detection rate ~ human settlements + roads + (1|camera.ID)	5	283.62	0	0.72	0.068	0.082
Asia badger detection rate ~ temperature + snow status + roads + (1|camera. ID)	6	136.51	0	0.41	0.065	0.255
Eurasian lynx detection rate ~ aspect + habitat types + (1|camera.ID)	7	110.57	0.000	1	0.020	0.143
Snow leopard detection rate ~ time + snow status + roads + (1|camera.ID)	6	385.22	0.000	0.82	0.053	0.231
Common leopard detection rate ~ temperature + snow status + altitude + habitat types + (1|camera.ID)	10	1693.88	0.000	1	0.044	0.210
Red fox detection rate ~ habitat types + (1|camera.ID)	6	1139.30	0.000	1	0.024	0.157
Red dog detection rate ~ roads + habitat types + (1|camera.ID)	7	82.53	0.000	1	0.072	0.026
yellow billed weasel detection rate ~ time + temperature + précipitation +habitat types + (1|Camera.ID)	9	47.08	0.000	1	4.518	2.125
Stone marten detection rate ~ water + (1|Camera.ID)	3	404.70	0.000	1	0.013	0.115

**Table 2:** Best model parameter estimates derived from LMMs (only considered positive or negative effects and non-effects removed from the tables). Accompanying these estimates are their standard errors (SE) and 95% confidence intervals (CI) for each individual mammalian species.

Variables	Fixed Effect	Estimate	Std. Error	Lower CI	Upper CI
Bule sheep	Intercept	2.492	2.721	0.196	0.303
	Aspect	0.119	6.055	0.013	0.018
Chines serow	Intercept	0.5	0.272	0.02	1.021
	NDVI	−0.018	0.069	−4.568	−0.031
	Scrub	0.148	0.279	0.39	6.791
Musk deer	Intercept	0.399	0.132	0.145	0.653
	Snow status (present)	−0.035	0.013	−0.088	−0.061
	Mixed forest	0.32	0.224	0.109	0.75
White lipped deer	Intercept	−1.526	2.002	−8.542	−4.407
	road	−2.207	2.767	−5.695	−2.148
	Scrub	2.631	2.062	2.714	6.077
Wolly hare	Intercept	−1.091	1.724	−7.537	−1.428
	Precipitation	−7.061	2.883	−1.362	−1.276
	Alpine meadow	2.549	1.693	5.855	7.807
	Evergreen coniferous forest	−3.942	1.759	−7.382	−4.921
	Water	−6.463	5.103	−7.495	−5.471
Tibetan pika	Intercept	−5.589	1.55	−8.748	−2.47
	Alpine meadow	2.623	1.194	2.746	4.953
	Scrub	0.47	0.598	0.099	3.351
Rhesus macaque	Intercept	1.096	0.613	−0.137	2.177
	Altitude	0.017	0.014	0.045	9.983
	NDVI	0.001	0.004	0.041	1.908
Himalayan marmot	Intercept	1.091	1.876	0.724	1.454
	Altitude	−6.528	4.256	−1.847	−0.014
Brown bear	Intercept	−1.059	0.282	−1.588	−0.525
	Temperature	−0.018	0.004	−1.048	−0.026
	Snow status present	−0.187	0.064	−6.222	−0.314
	Mixed forest	0.278	0.437	0.546	1.109
Grey wolf	Intercept	−6.09	1.11	−8.312	−3.858
	Human settlements	−7.064	5.672	−3.84	−1.853
	roads	−4.312	8.269	−1.983	−1.137
Asian badger	Intercept	−8.624	1.724	−5.304	−1.193
	Temperature	−5.158	8.117	−2.145	−1.043
	Snow status (present)	−1.622	1.654	−3.356	−3.102
Leopard cat	(Intercept)	−6.864	5.759	−0.813	−0.563
	Slope	−4.033	1.006	−0.024	−0.016
Eurasian lynx	Intercept	−7.721	1.498	−1.025	−0.52
	Aspect	−1.482	8.926	−2.703	−0.029
	Scrub	0.314	0.915	0.195	4.536
Snow leopard	Intercept	−5.727	9.735	−7.621	−3.835
	Snow status (on)	3.807	6.023	0.55	8.062
	roads	−1.155	7.146	−2.555	−2.383
Common leopard	Intercept	0.891	0.232	0.407	1.333
	Temperature	−0.001	0.016	−1.339	−0.004
	altitude	−0.005	0.045	−4.437	−0.001
	mixed forest	0.187	0.227	0.24	6.185
	Evergreen coniferous forest	0.021	0.162	0.291	3.286
Red fox	Intercept	0.938	0.15	0.648	1.226
	Alpine meadow	−0.146	0.159	−0.451	−0.159
	Scrub	0.193	0.155	0.104	0.491
Red dog	Intercept	−1.281	2.257	−8.574	−1.707
	roads	−2.565	1.338	−5.081	−4.33
	Evergreen coniferous forest	−6.14	2.039	−9.99	−2.209
	Scrub	−4.845	2.347	−9.287	−3.81
Yellow billed weasel	Intercept	−7.939	2.68	2.949	12.929
	Precipitation	−0.011	0.004	−0.019	−0.003
	Evergreen coniferous forest	0.086	0.099	0.099	0.271
Stone marten	Intercept	−8.953	6.37	−7.696	−1.019
	water	−2.654	1.337	−5.328	−1.556

## Data Availability

The datasets used in this study are available from the corresponding author on reasonable request.

## Supplementary Materials

The following supporting information can be downloaded at: https://www.mdpi.com/article/10.3390/biology15110862/s1, Figure S1: Mean species richness and mean species evenness across four habitat types (presence vs. Absence of snow) (25 mammalian species, Tibetan region); Figure S2: Interaction between mammalian species and data detection rates of predator-prey pairs, analyzed using language models (lms). The shaded line illustrates the interactions between specific species pairs; Table S1: Mammalian species in the study site and their status in China; Table S2: Data on mammalian species diversity, including the sample size (n), observed species richness (S. obs), and comparisons of diversity indices using Hill numbers. The parameter ‘q’ determines the type of diversity index: q = 0 corresponds to species richness; q = 1 reflects the Shannon diversity index; and q = 2 represents the Simpson diversity index. Increasing values of ‘q’ diminish the impact of rare species on the overall diversity estimate. The table also reports the standard error (S.E.) of sample coverage, along with the estimated values (est) and their 95% confidence intervals (lower confidence limit, LCL; upper confidence limit, UCL); Table S3: Data on habitat factors, including the sample size (n), observed species richness (S.obs), and comparisons of diversity indices using Hill numbers. The parameter ‘q’ determines the type of diversity index: q = 0 corresponds to species richness; q = 1 reflects the Shannon diversity index; and q = 2 represents the Simpson diversity index. Increasing values of ‘q’ diminish the impact of rare species on the overall diversity estimate. The table also reports the standard error (S.E.) of sample coverage, along with the estimated values (est) and their 95% confidence intervals (lower confidence limit, LCL; upper confidence limit, UCL).
