# Supplementary material for: Unraveling Mammalian Biodiversity in a Non-Protected Area in Tibet: Community Diversity, Species Interactions and Conservation Imperatives
Source: Biology (Basel). 2026 May 30;15(11):862. doi: 10.3390/biology15110862 (PMC13255578; doi:10.3390/biology15110862)
Supplement: Supplementary file 1 [file biology-15-00862-s001.zip › biology-4286915-supplementary.pdf]

Table S1. Mammalians species in study site and their status in China.

**Common Name**	**Scientific Name**	**Genus**	**Family**	**Conservation Status in China**
White-lipped deer	*Cervus albirostris*	Cervus	Cervidae	National First-Class Protected Species
Common leopard	*Panthera pardus*	Panthera	Felidae	National First-Class Protected Species
Leopard cat	*Prionailurus bengalensis*	Prionailurus	Felidae	National Second-Class Protected Species
Dhole (red dog)	*Cuon alpinus*	Cuon	Canidae	National First-Class Protected Species
Red fox	*Vulpes vulpes*	Vulpes	Canidae	National Key Protected (Ecological/Scientific)
Plateau pika	*Ochotona curzoniae*	Ochotona	Ochotonidae	National Key Protected (Ecological/Scientific)
Asian badger	*Meles leucurus*	Meles	Mustelidae	National Key Protected (Ecological/Scientific)
Yellow-throated marten	*Martes flavigula*	Martes	Mustelidae	National Second-Class Protected Species
Himalayan weasel	*Mustela sibirica*	Mustela	Mustelidae	National Key Protected (Ecological/Scientific)
Woolly hare	*Lepus oiostolus*	Lepus	Leporidae	National Key Protected (Ecological/Scientific)
Gray wolf	*Canis lupus*	Canis	Canidae	National Second-Class Protected Species
Alpine musk deer	*Moschus chrysogaster*	Moschus	Moschidae	National First-Class Protected Species
Rhesus macaque	*Macaca mulatta*	Macaca	Cercopithecidae	National Second-Class Protected Species
Eurasian lynx	*Lynx lynx*	Lynx	Felidae	National Second-Class Protected Species

**Common Name**	**Scientific Name**	**Genus**	**Family**	**Conservation Status in China**
Stone marten	*Martes foina*	Martes	Mustelidae	National Key Protected (Ecological/Scientific)
Mountain weasel	*Mustela altaica*	Mustela	Mustelidae	National Second-Class Protected Species
Himalayan marmot	*Marmota himalayana*	Marmota	Sciuridae	National Key Protected (Ecological/Scientific)
Snow leopard	*Panthera uncia*	Panthera	Felidae	National First-Class Protected Species
Blue sheep	*Pseudois nayaur*	Pseudois	Bovidae	National Second-Class Protected Species
Wild boar	*Sus scrofa*	Sus	Suidae	National Key Protected (Ecological/Scientific)
Tibetan fox	*Vulpes ferrilata*	Vulpes	Canidae	National Key Protected (Ecological/Scientific)
Hog badger	*Arctonyx collaris*	Arctonyx	Mustelidae	National Key Protected (Ecological/Scientific)
Chinese serow	*Capricornis milneedwardsii*	Capricornis	Bovidae	National First-Class Protected Species
Brown bear	*Ursus arctos*	Ursus	Ursidae	National Second-Class Protected Species

Table S2 Data on mammalian species diversity, including the sample size (n), observed species richness (S. obs), and comparisons of diversity indices using Hill numbers. The parameter 'q' determines the type of diversity index: q = 0 corresponds to species richness; q = 1 reflects the Shannon diversity index; and q = 2 represents the Simpson diversity index. Increasing values of 'q' diminish the impact of rare species on the overall diversity estimate. The table also reports the standard error (S.E.) of sample coverage, along with the estimated values (est) and their 95% confidence intervals (lower confidence limit, LCL; upper confidence limit, UCL).

Species name	n	q=0(Obs)	est	S.e	LCL	UCL	q=1(obs)	est	S.E	LCL	UCL	q=2(obs)	estim	S.E	LCL	UCL

AMD	990	26.00	26.00	0.85	26.00	27.66	17.04	17.26	0.52	16.23	18.28	12.61	12.76	0.63	11.53	14.07
BB	337	12.00	12.49	1.62	12.00	15.68	5.69	5.79	0.36	5.07	6.51	3.72	3.75	0.28	3.18	4.31
CS	742	22.00	24.99	3.06	22.00	31.00	11.13	11.32	0.46	10.40	12.24	7.86	7.93	0.43	7.07	8.79
GW	513	11.00	11.00	1.26	11.00	13.48	5.32	5.38	0.25	4.88	5.87	3.68	3.70	0.23	3.25	4.16
HB	333	14.00	16.99	1.75	14.00	20.42	8.40	8.62	0.38	7.88	9.37	6.79	6.91	0.38	6.15	7.68
Leo	1029	24.00	26.99	2.43	24.00	31.76	11.71	11.87	0.31	11.26	12.48	8.33	8.39	0.27	7.84	8.94
MARM	72	8.00	10.21	4.35	8.00	18.75	2.50	2.69	0.41	1.88	3.51	1.62	1.64	0.18	1.27	2.07
RD	107	6.00	6.00	0.64	6.00	7.25	3.26	3.34	0.30	2.74	3.94	2.39	2.42	0.26	1.91	2.94
RF	1237	25.00	26.99	3.48	25.00	33.82	11.55	11.67	0.34	10.99	12.35	7.66	7.70	0.33	7.04	8.35
SLeo	362	16.00	17.99	2.706	16.00	23.29	8.73	8.95	0.38	8.19	9.71	6.60	6.70	0.40	5.90	7.51
SMAR	532	21.00	21.49	1.75	21.00	24.93	12.15	12.39	0.43	11.53	13.25	8.85	8.99	0.47	8.06	9.92
WB	14	2.00	2.00	0.01	2.00	2.00	2.00	2.07	0.08	1.90	2.24	2.00	2.16	0.15	1.85	2.47
WH	830	23.00	23.00	0.44	23.00	23.87	13.81	14.07	0.38	13.25	14.76	10.55	10.68	0.40	9.88	11.47
YBW	68	6.00	6.49	1.51	6.00	9.45	3.13	3.27	0.40	2.47	4.07	2.20	2.24	0.30	1.64	2.84
HW	29	3.00	3.00	0.25	3.00	3.49	2.04	2.12	0.27	1.58	2.66	1.65	1.69	0.26	1.179	2.20
LC	443	17.00	18.99	3.33	17.00	25.54	9.02	9.21	0.39	8.45	9.98	6.54	6.62	0.37	5.89	7.36
MAC	195	11.00	15.47	4.85	11.00	24.99	6.33	6.58	0.38	5.83	7.329	5.32	5.44	0.29	4.855	6.02
MW	428	11.00	11.49	1.45	11.00	14.34	4.40	4.46	0.25	3.96	4.95	3.11	3.13	0.18	2.77	3.49
PP	422	18.00	18.16	1.97	18.00	22.04	10.41	10.64	0.50	9.65	11.62	8.07	8.21	0.46	7.30	9.12
BS	1059	23.00	27.49	6.51	23.00	40.26	7.29	7.38	0.30	6.80	7.97	4.30	4.32	0.20	3.92	4.71
WLD	51	6.00	6.00	0.40	6.00	6.79	4.34	4.56	0.41	3.75	5.37	3.43	3.61	0.48	2.65	4.57
AB	100	5.00	6.98	2.17	5.00	11.23	1.83	1.90	0.19	1.52	2.28	1.44	1.45	0.12	1.20	1.69
YTM	9	3.00	3.88	0.77	3.00	5.41	1.98	2.42	0.51	1.41	3.43	1.58	1.71	0.45	0.83	2.59
EL	298	7.00	7.00	0.14	7.00	7.27	4.82	4.87	0.19	4.48	5.26	4.02	4.06	0.21	3.65	4.48
TFX	2	2.00	2.50	0.62	2.00	3.72	2.00	3.37	0.98	1.43	5.31	2.00	2.01	0.22	1.33	3.11

Table S3 This table presents data habitat factors, including the sample size (n), observed species richness (S.obs), and comparisons of diversity indices using Hill numbers. The parameter 'q' determines the type of diversity index: q = 0 corresponds to species richness; q = 1 reflects the Shannon diversity index; and q = 2 represents the Simpson diversity index. Increasing values of 'q' diminish the impact of rare species on the overall diversity estimate. The table also reports the standard error (S.E.) of sample coverage, along with the estimated values (est) and their 95% confidence intervals (lower confidence limit, LCL; upper confidence limit, UCL).

Habitats	n	q=0(Obs)	est	S.e	LCL	UCL	q=1(obs)	est	S.E	LCL	UCL	q=2(obs)	estim	S.e	LCL	UCL
Alpine meadow	6326	218.00	235.63	9.547	218.00	254.34	96.08	97.97	1.38	95.27	100.68	54.18	54.64	1.24	52.20	57.09
Evergreen coniferous forest	8257	215.00	239.99	12.99	215.00	265.47	109.92	111.58	1.42	108.79	114.37	69.96	39.97	8.42	25.00	56.48
Mixed forest	597	25.00	39.97	8.42	25.00	56.48	12.30	12.70	0.52	11.66	13.73	7.77	7.86	0.501	6.88	8.84
Scrub	9684	293.00	326.99	14.27	299.01	354.97	137.83	140.29	1.50	137.35	143.23	85.62	86.38	1.67	83.09	89.66
Absent	-	320.00	360.26	13.74	333.32	387.19	148.88	151.66	1.53	148.65	154.67	92.17	93.00	1.74	89.57	96.43
present	-	2.00	2.00	0.35	2.00	2.70	2.00	2.30	0.42	0.11	2.00	2.00	3.00	0.68	0.11	2.11
Elevation	-	11.75	11.70	0.19	0.18	0.197	50.32	49.24	0.66	0.65	0.67	42.71	40.98	0.77	0.76	0.77
Slope	-	102.17	100.40	0.83	0.83	0.844	43.31	41.53	0.78	0.77	0.79	43.85	42.03	0.79	0.79	0.80
Aspect	-	78.37	76.19	0.90	0.90	0.908	78.77	76.57	0.90	0.90	0.91	7.66	7.70	0.33	7.04	8.35
Humans	-	1800	17.91	3.70	16.00	23.29	8.73	8.95	0.38	8.19	9.71	6.60	6.70	0.40	5.90	7.51
Roads	-	212.00	212.49	0.75	111.55	113.51	12.15	12.39	0.43	9	10.77	8.85	8.99	0.47	22.00	33.00
Streams	-	233.00	222.00	0.70	12.00	12.00	12.00	12.06	8.08	1.96	22.24	12.00	12.16	80.15	11.85	15.55

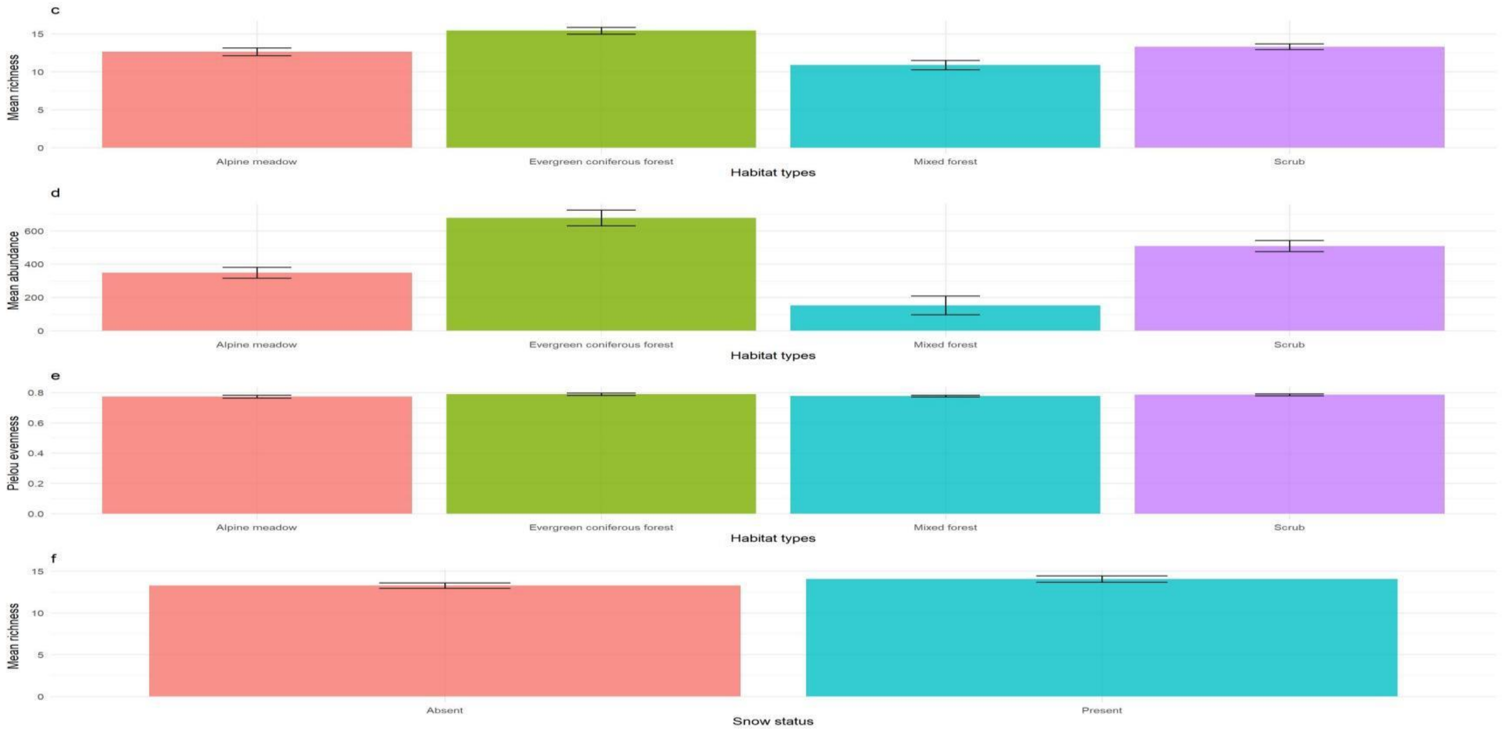

Figure S1: Mean species richness and mean species evenness across four habitat types (presence vs. Absence of snow) (25 mammalian species, tibetan region)

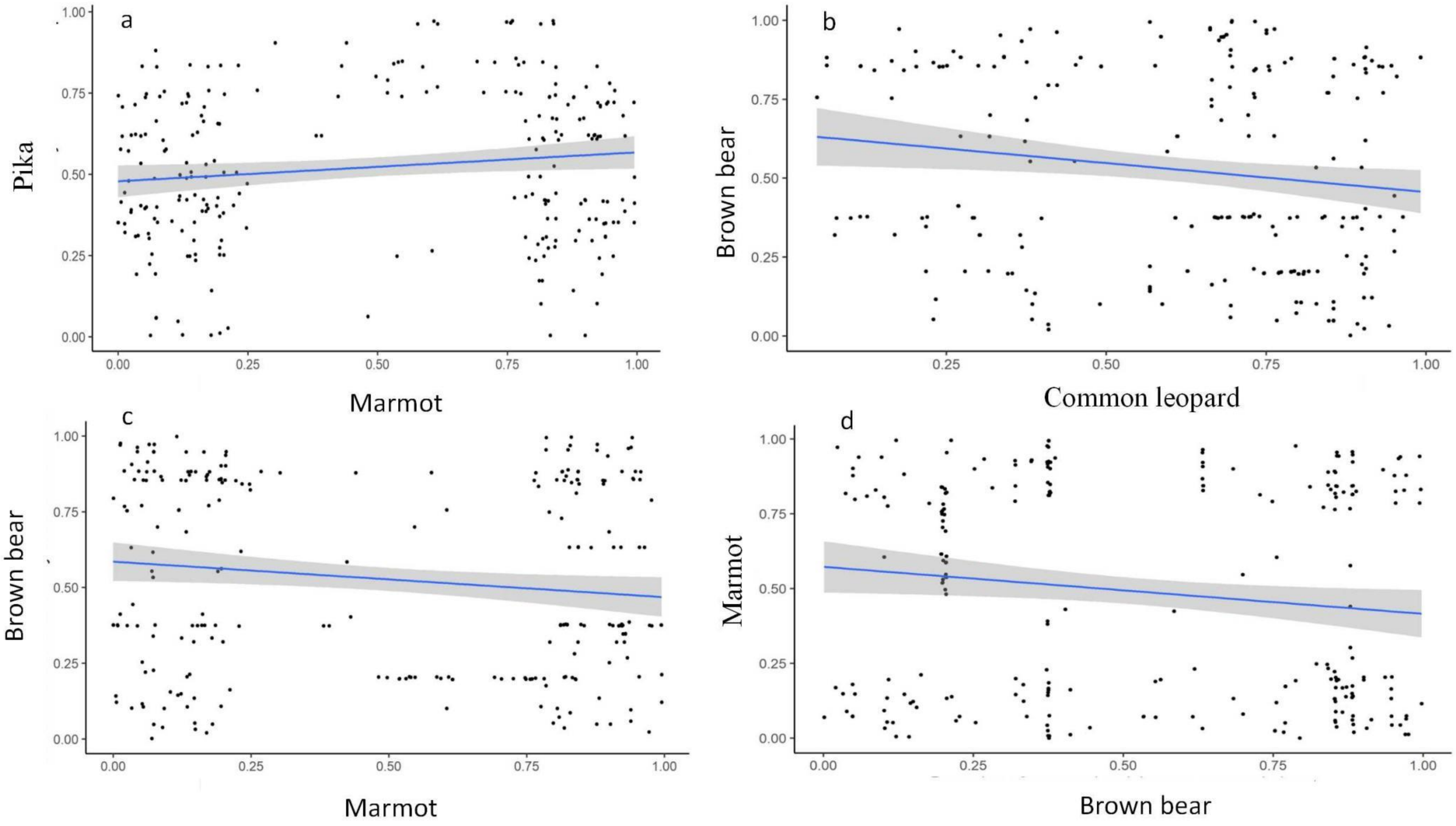

a
Pika
Marmot
b
Brown bear
Common leopard
c
Brown bear
Marmot
d
Marmot
Brown bear

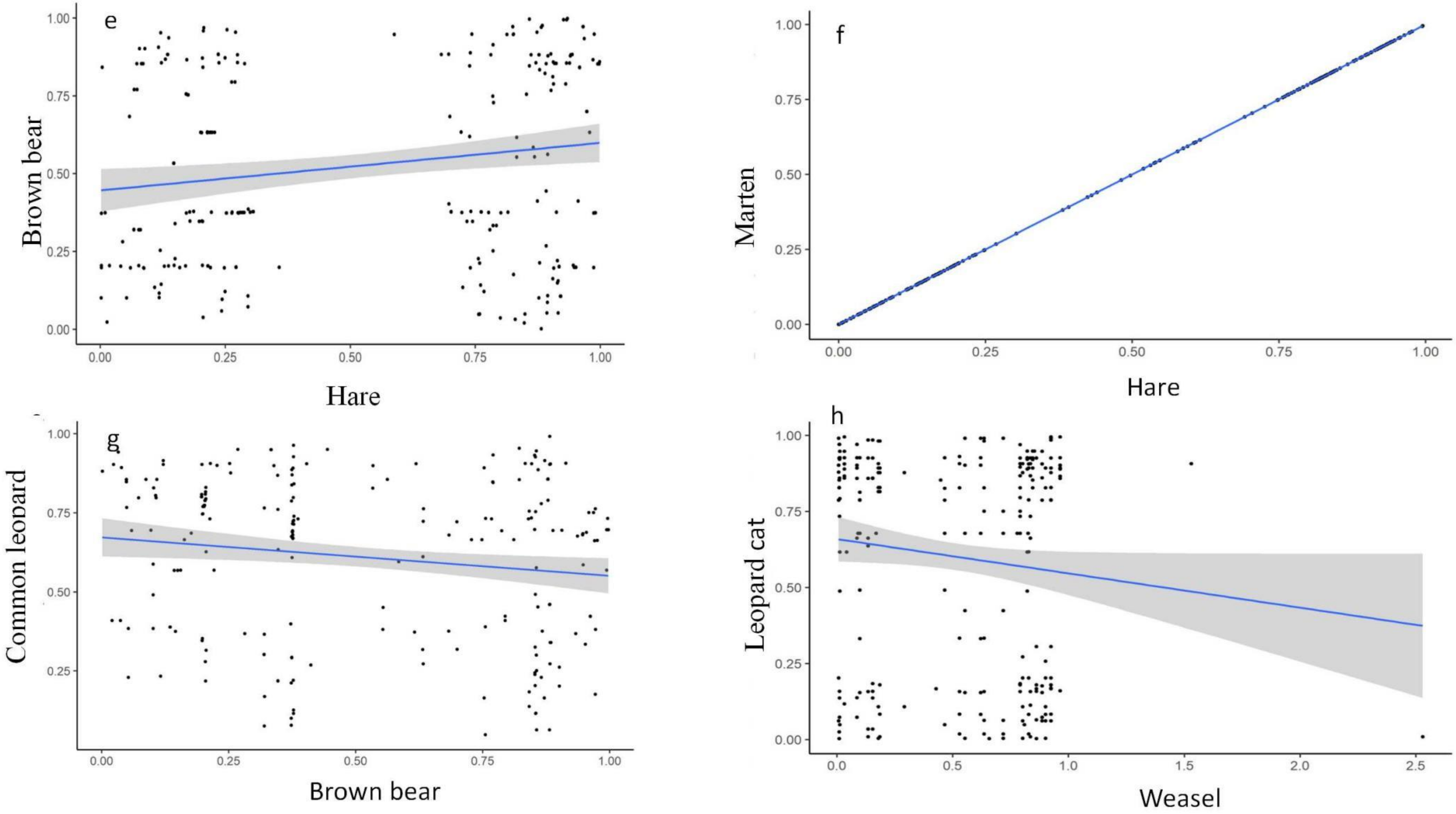
e
Brown bear
1.00
0.75
0.50
0.25
0.00
0.00
0.25
0.50
0.75
1.00
Hare
f
Marten
1.00
0.75
0.50
0.25
0.00
0.00
0.25
0.50
0.75
1.00
Hare
g
Common leopard
1.00
0.75
0.50
0.25
0.00
0.25
0.50
0.75
1.00
Brown bear
h
Leopard cat
1.00
0.75
0.50
0.25
0.00
0.0
0.5
1.0
1.5
2.0
2.5
Weasel

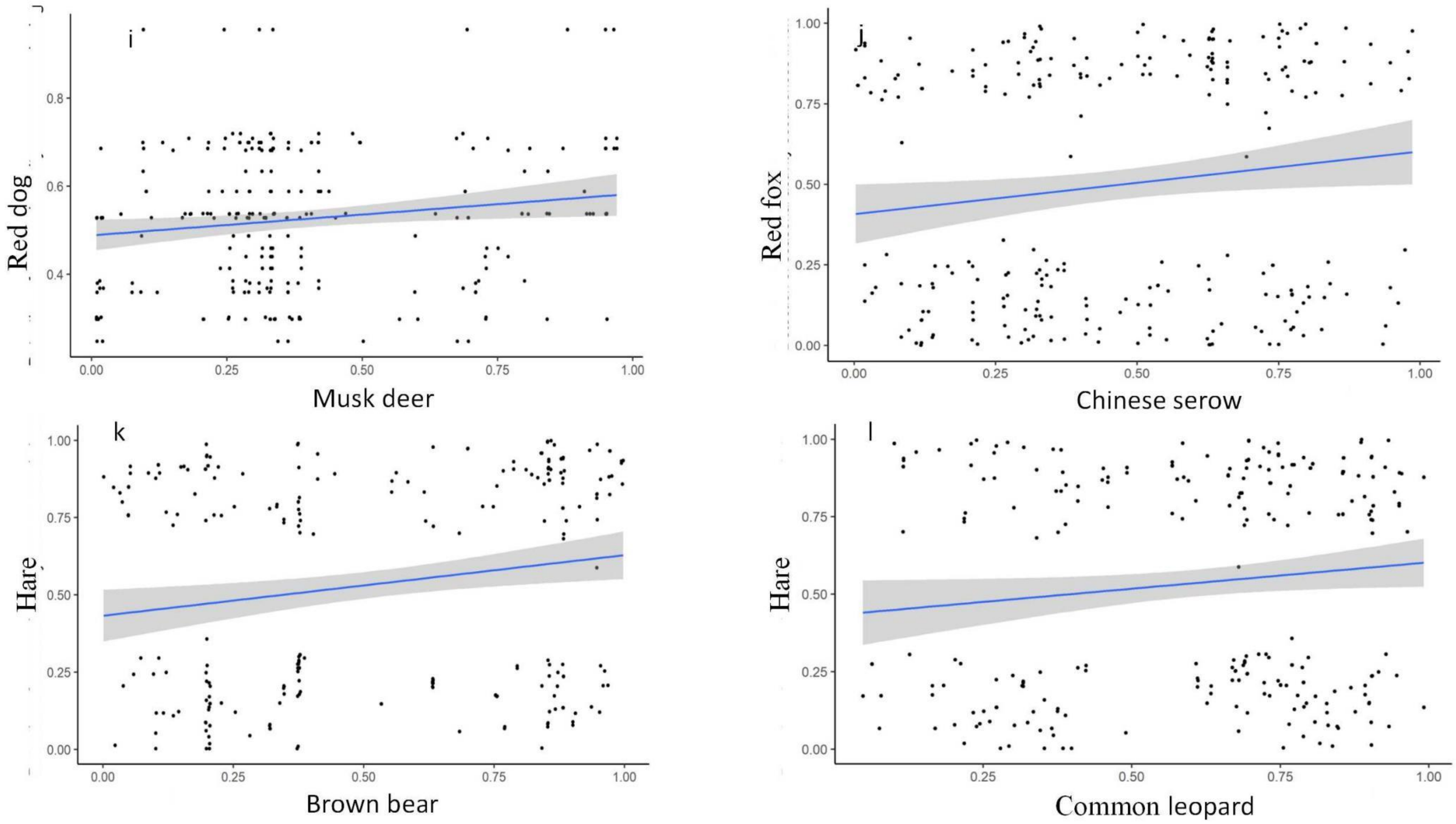

i
Red dog
Musk deer
j
Red fox
Chinese serow
k
Hare
Brown bear
l
Hare
Common leopard

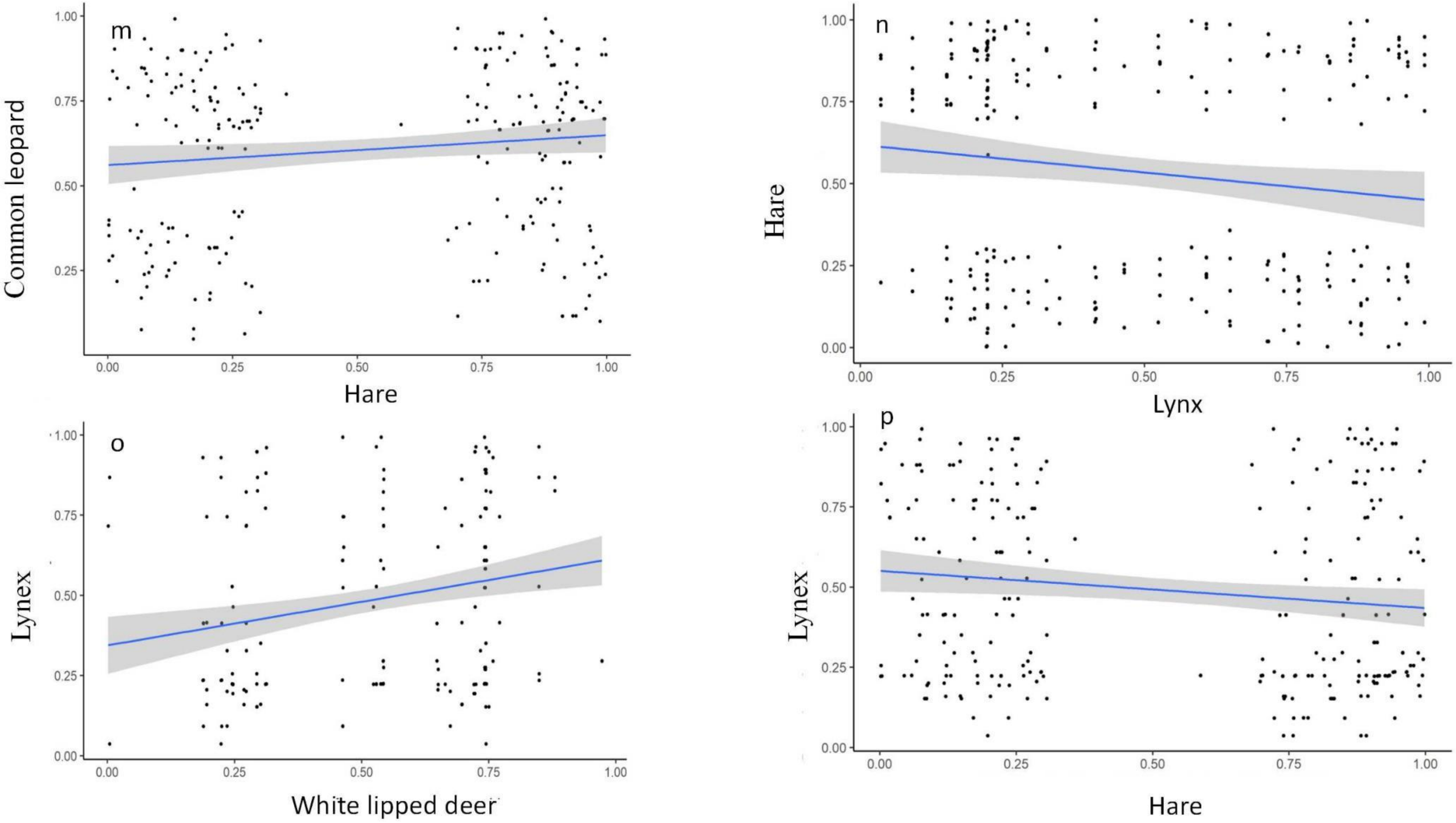
m
Common leopard
Hare
n
Hare
Lynx
o
Lynex
White lipped deer
p
Lynex
Hare
0.00
0.25
0.50
0.75
1.00

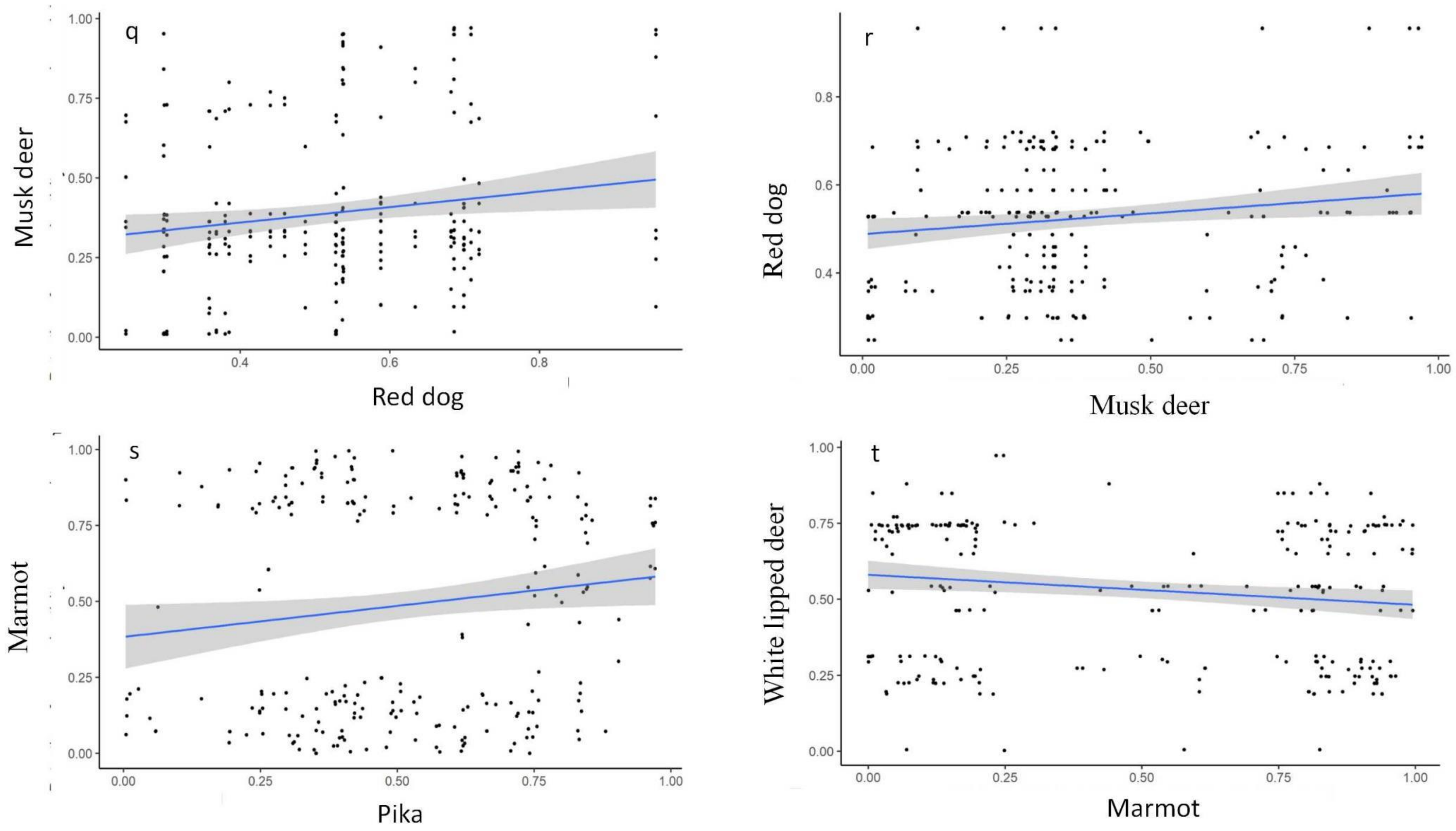
q
Musk deer
Red dog
r
Red dog
Musk deer
s
Marmot
Pika
t
White lipped deer
Marmot

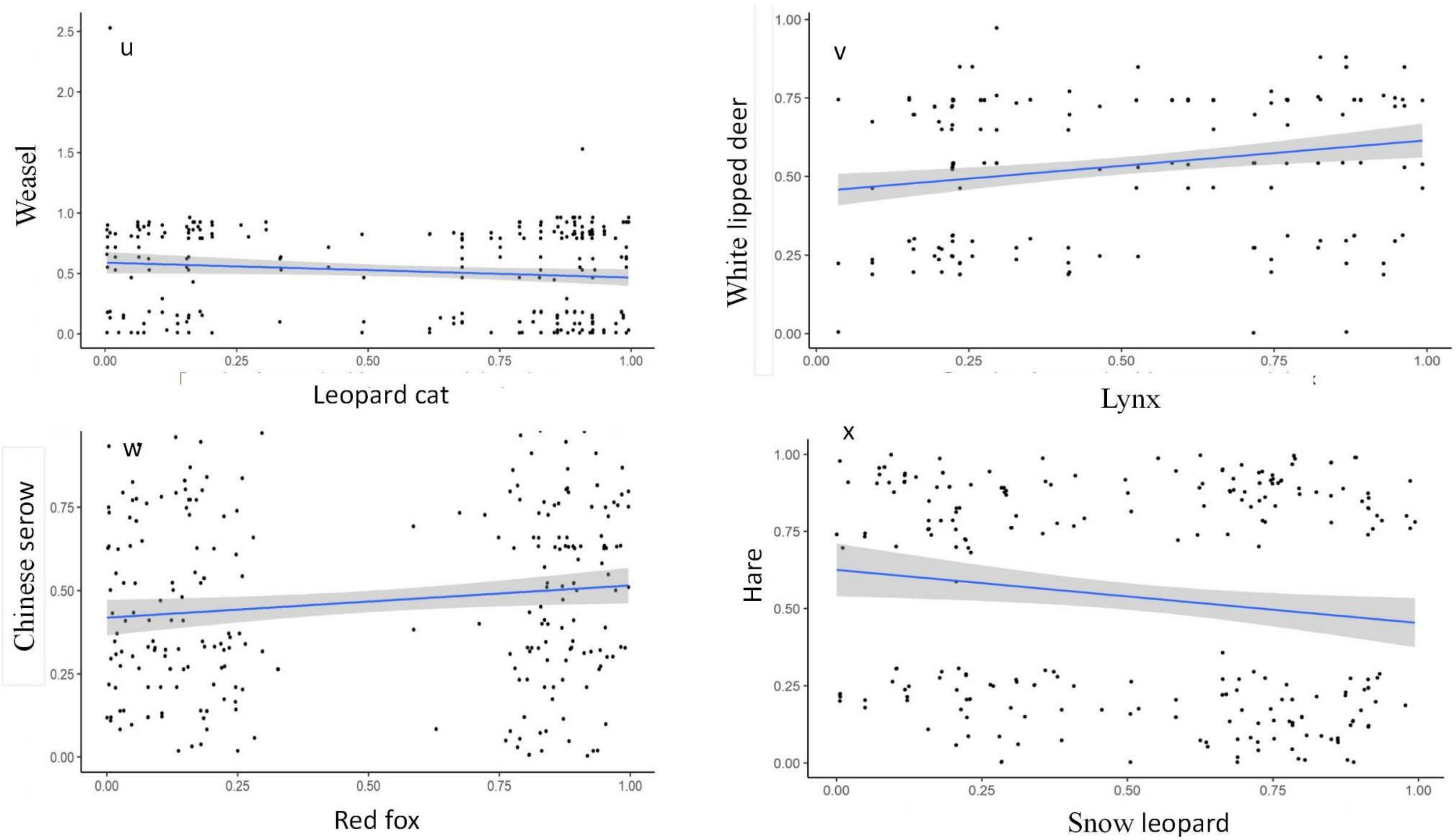

u
Weasel
Leopard cat
v
White lipped deer
Lynx
w
Chinese serow
Red fox
x
Hare
Snow leopard

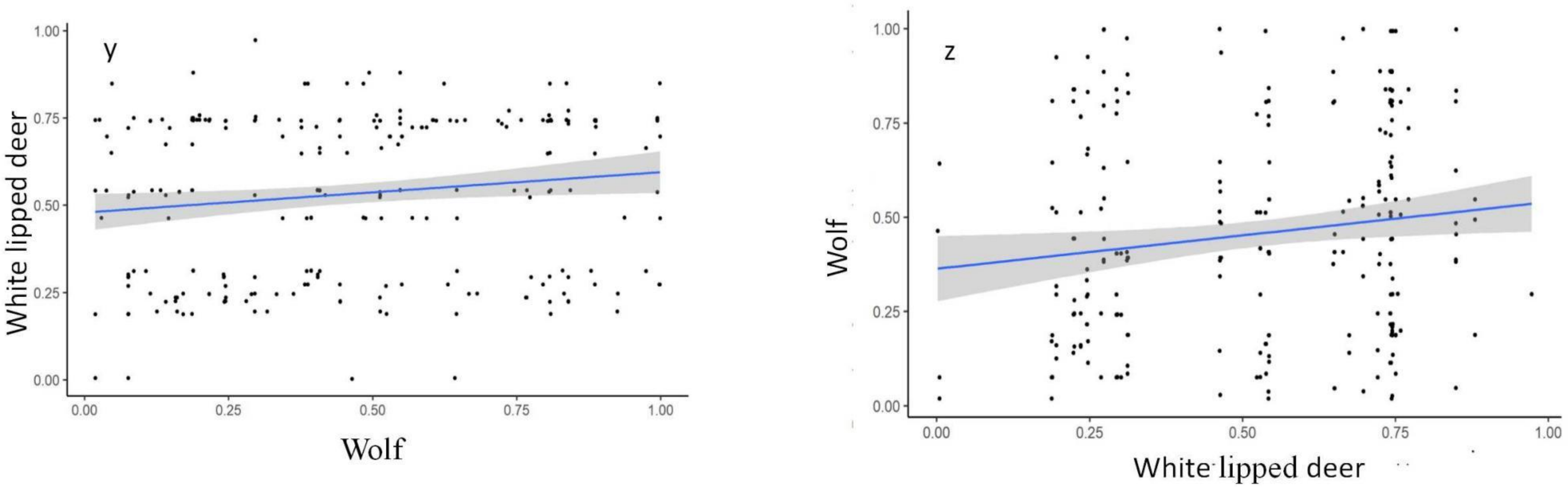

Figure S2. Interaction between mammalian species and data detection rates of predator-prey pairs, analyzed using language models (lms). The shaded line illustrates the interactions between specific species pairs.
